# *In Vitro* Effects of Extracellular Vesicles from Adipose Tissue-Derived Stem Cells on the Growth and Metastasis of Cultured Breast Cancer Cells via Downregulation of Interleukin-6 Expression and the Microtubule Network

**DOI:** 10.3390/biology15010052

**Published:** 2025-12-28

**Authors:** Huyen Thi La, Hai Manh Tran, Phuc Minh Thi Le, Huyen Thi Ngo, Hanh Hong Hoang, Da Thi Nguyen, Linh Thuy Nguyen, Nghia Trong Nguyen, Lien Ha Thi Nghiem, Van Hanh Nguyen, Long Hoang Nguyen, Van Ngoc Bui, Nam Trung Nguyen, Ha Hoang Chu

**Affiliations:** 1 Institute of Biology, Vietnam Academy of Science and Technology, Hanoi 100000, Vietnam; huyenla.ibt@gmail.com (H.T.L.);; 2 Vietnam Academy of Science and Technology, Graduate University of Science and Technology, Hanoi 100000, Vietnam; 3 Institute of Physics, Vietnam Academy of Science and Technology, Hanoi 118000, Vietnam; 4 Faculty of Agricultural Technology, VNU University of Engineering and Technology, 144 Xuan Thuy, Cau Giay, Hanoi 100000, Vietnam; 5 Institute for Advanced Study in Technology, Ton Duc Thang University, Ho Chi Minh City 700000, Vietnam; 6 Faculty of Pharmacy, Ton Duc Thang University, Ho Chi Minh City 700000, Vietnam

**Keywords:** ADSC-derived extracellular vesicles, MCF-7 cells, breast cancer, IL-6/STAT3 signaling, microtubule disruption

## Abstract

Breast cancer remains the most common malignancy worldwide and the leading cause of cancer-related mortality. Recently, extracellular vesicles (EVs) derived from adipose tissue-derived stem cells (ADSCs) have attracted increasing attention for their potential to modulate inflammatory signaling and influence tumor cell behavior. This in vitro study was designed to investigate the effects of ADSC-EVs on MCF-7 breast cancer cells. EVs were isolated from ADSC culture supernatants and applied to MCF-7 cells at concentrations ranging from 0 to 80% (*v*/*v*). Cell viability, migration, and expression of IL-6/STAT3 pathway-related genes were evaluated using MTT, scratch assays, and qRT-PCR. Statistical analysis was performed using one-way ANOVA followed by Tukey’s post hoc test, with significance set at *p* < 0.05. The results showed that 20% EV treatment markedly inhibited MCF-7 cell activity, significantly reducing viability and almost completely blocking migration, with wound closure rates of 35.4% ± 3.80 at 24 h and 47.6% ± 4.2 at 48 h, compared with 48% ± 4.6 and 67% ± 4.2 in the control group, respectively. Notably, expression levels of IL-6, IL-6RST, and STAT3 were significantly downregulated (fold changes 0.155 ± 0.02 and 0.258 ± 0.012, *p* < 0.01), accompanied by severe disruption of the microtubule network. Immunofluorescence imaging revealed a disorganized microtubule architecture and irregular filament distribution in EV-treated cells, corresponding with decreased expression of TubA1 and CALR genes. These findings indicate that ADSC-EVs not only suppress IL-6/STAT3 inflammatory signaling but also destabilize the intracellular microtubule system, collectively contributing to the inhibition of MCF-7 breast cancer cell migration and survival. This provides an important molecular basis for developing novel EV-based therapeutic strategies in breast cancer treatment.

## Simple Summary

Breast cancer is the most common cancer in women worldwide and remains a major cause of death. Scientists are exploring new treatments that go beyond surgery and chemotherapy. This study looked at tiny natural particles called extracellular vesicles, released by fat-derived stem cells, to see how they affect breast cancer cells in the lab. The extracellular vesicles were isolated with high purity and predominantly fell within the nanoscale size range, with an average diameter of 177.1 ± 78.7 nm. When breast cancer cells were treated with these vesicles, their growth and movement slowed, especially at a moderate concentration. The treatment also reduced the levels of molecules linked to inflammation and cancer progression, such as IL-6 and STAT3, and disrupted the internal skeleton that helps cells divide and spread. Under the microscope, cancer cells exposed to the vesicles showed disorganized structures and less stability. These results suggest that substances naturally produced by stem cells might help slow or stop cancer cell growth by blocking harmful signaling and weakening the cell’s internal framework. This research could open the way for new, cell-free treatments that use the body’s own biological materials to fight breast cancer safely and effectively.

## 1. Introduction

Breast cancer is the most frequently diagnosed malignancy worldwide and ranks as the fifth leading cause of cancer-related mortality overall. Among women, it is the most common cancer in 157 of 185 countries. In 2022, approximately 2.3 million new cases were diagnosed globally, leading to an estimated 670,000 deaths [1]. Despite the decline in mortality due to advancements in early detection and treatment, the global incidence of breast cancer has continued to rise [2]. While non-surgical treatment options including radiotherapy and chemotherapy are available, lumpectomy has remained the primary therapeutic approach [3,4]. In cases of advanced disease, mastectomy is often required, thereby increasing the demand for breast reconstruction procedures [5,6].

Autologous fat grafting (AFG), also referred to as autologous fat transfer, has emerged as a widely used technique in both cosmetic and reconstructive surgery. Adipose tissue, which can be harvested through minimally invasive methods, is regarded as a rich source of adipose-derived stem cells (ADSCs). Due to their immunomodulatory, angiogenic, and proliferative properties, ADSCs have been considered a promising candidate for applications in regenerative medicine [7,8,9]. A population of undifferentiated, multipotent, and self-renewing progenitor cells was first isolated from white adipose tissue by Zuk et al. (2002) [7], and these cells were found to resemble bone marrow-derived mesenchymal stem cells (BM-MSCs) in both morphology and phenotype. Additionally, they were shown to influence epithelial–mesenchymal transition (EMT) and contribute to invasive cellular behavior [7].

ADSCs have demonstrated the ability to differentiate into multiple mesenchymal lineages, including adipocytes, osteoblasts, and hepatocytes. Their therapeutic potential is attributed to the secretion of cytokines, chemokines, and growth factors, which exert anti-apoptotic, anti-angiogenic, anti-inflammatory, and immunomodulatory effects. Furthermore, ADSC harvesting is less invasive and yields a significantly greater number of cells—approximately 1000 times more—than BM-MSCs and cord blood-derived MSCs. Compared to BM-MSCs, ADSCs also exhibit enhanced proliferative capacity, extended lifespan, shortened doubling time, and delayed senescence in vitro. These characteristics have rendered them highly suitable for regenerative applications. In clinical settings, stem cells may be administered directly into damaged tissues or used in a cell-free approach through extracellular vesicles (EVs), which are known to mediate therapeutic effects via paracrine mechanisms [10,11].

Extracellular vesicles (EVs) are nanoscale particles encapsulated by lipid bilayers and secreted by cells. They carry diverse biomolecules from their cells of origin but lack self-replicative ability [12,13]. The cargo contained within EVs includes lipids, proteins, macromolecular complexes, DNA, and various RNA types, such as siRNA, miRNA, lncRNA, and circRNA [14,15,16,17]. In some instances, intact organelles have also been found within EVs. Techniques such as nucleic acid sequencing and mass spectrometry have facilitated the detailed characterization of EV cargoes, providing insight into the physiological and metabolic status of donor cells. The specificity and molecular diversity of these cargoes have positioned EVs as promising biomarkers for disease diagnosis and monitoring [18].

Intercellular communication, especially between different or similar cell types, is often mediated through paracrine signaling involving the secretion of EVs and bioactive molecules [17]. EVs are categorized primarily into microvesicles and exosomes, based on their size and origin. Microvesicles (100–1000 nm), also termed ectosomes, are formed by outward budding of the plasma membrane, while exosomes (30–100 nm) are generated through endosomal pathways and released via exocytosis [19]. In regenerative medicine, ADSC-derived EVs (ADSC-EVs) have shown promising results in diverse medical applications, including skin wound healing [20,21,22], peripheral artery disease [23], myocardial infarction [24,25,26], diabetic nephropathy [27,28,29], fat graft survival [30,31,32,33], bone regeneration [34,35], cartilage regeneration [36,37], tendinopathy and tendon healing [38,39,40,41,42,43,44,45,46], the peripheral nervous system [47,48,49,50], acute lung injury [51,52], hepatic ischemia–reperfusion injury [53], skeletal muscle injury [54,55], thin endometrium-induced infertility [56], and type 2 diabetes mellitus [57].

Recent studies have shown that extracellular vesicles derived from adipose tissue-derived stem cells (ADSC-EVs) exert potent anti-tumor effects in breast cancer models by regulating key cellular processes such as proliferation, migration, and apoptosis. Li et al. demonstrated that ADSC-EVs significantly suppressed tumor growth and promoted apoptotic activity in murine breast cancer models [58]. Similarly, Pagani et al. reported that ADSC-EVs not only affected primary tumor cells but also modulated interactions within the tumor microenvironment and metastatic niches, underscoring their potential as multifaceted therapeutic agents [59].

Despite these benefits, concerns persist regarding the oncological safety of autologous fat grafting. As ADSCs promote angiogenesis and cellular proliferation through paracrine signaling, a potential risk of tumor recurrence has been raised [14]. Interleukin-6 (IL-6), a multifunctional cytokine, plays a key role in immune regulation, inflammation, and cell proliferation [60]. Its downregulation has been associated with inhibited growth of MCF-7 breast cancer cells [61]. Recent findings by Wareham et al. (2021) [62] indicated that IL-6 deficiency impairs anterograde axonal transport in vivo, with associated alterations in microtubule structure and function. The IL-6-dependent phenotype was attributed to protein interactions between STAT3 and stathmin, which stabilize microtubules—a mechanism potentially conserved across various systems, including retinal ganglion cells and tumor cells. Given these conflicting reports, further investigation is warranted to clarify the role of ADSC-EVs in breast cancer biology. Therefore, the objective of this study was to evaluate the effects of ADSC-derived extracellular vesicles on MCF-7 breast cancer cells, with a focus on cell viability, migration, IL-6/STAT3 signaling, and cytoskeletal structure.

Although extracellular vesicles derived from adipose tissue-derived stem cells (ADSC-EVs) have been extensively investigated for their regenerative and immunomodulatory properties, their specific role in breast cancer progression remains insufficiently understood. In particular, the effects of ADSC-EVs on the IL-6/STAT3 signaling pathway and microtubule organization—two key regulators of tumor growth and metastasis—have not been systematically explored. The present study aims to fill this gap by elucidating the molecular and structural impacts of ADSC-EVs on MCF-7 breast cancer cells.

## 2. Materials and Methods

### 2.1. Materials

**Figure 1:**
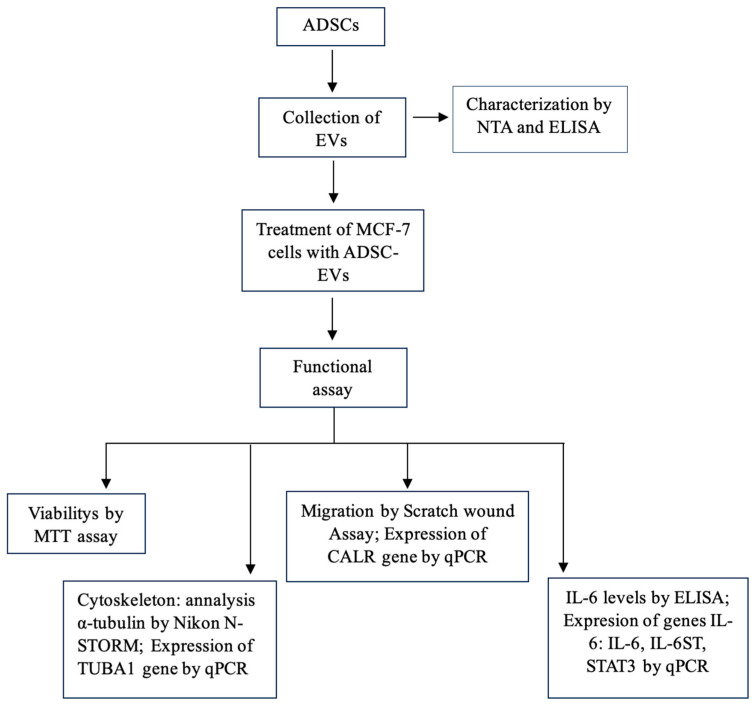
Schematic workflow illustrating the experimental sequence.

The MCF-7 breast cancer cell line (ATCC^®^ HTB-22™) and adipose-derived stem cells (ADSCs; PT-5006, Lonza, Walkersville, MD, USA) were maintained at the Animal Cell Technology Laboratory, Institute of Biotechnology, Vietnam Academy of Science and Technology. Both cell lines were maintained in their respective growth media under Biosafety Level 2 (BSL-2) conditions and subcultured following the manufacturer’s guidelines to ensure viability and authentication; ADSCs were used at passages 3–6. The study relied solely on commercially available cell lines, and therefore involved no human participants or animal experimentation. The schematic workflow is illustrated in Figure 1.

### 2.2. Method

#### 2.2.1. Culture of MCF-7 Cells

MCF-7 cells were cultured in Dulbecco’s Modified Eagle Medium (DMEM) (PAN-Biotech, GmbH, Aidenbach, Germany), supplemented with 10% fetal bovine serum (FBS) (Sigma-Aldrich, St. Louis, MO, USA) and 1% penicillin–streptomycin (PS) (Gibco-Thermo Scientific, Waltham, MA, USA). The cultures were incubated at 37 °C in a humidified atmosphere containing 5% CO_2_.

#### 2.2.2. Culture of ADSCs and Collection of Extracellular Vesicles

ADSCs were cultured in DMEM/F12 medium supplemented with 0.5 ng/mL fibroblast growth factor (FGF) (Gibco, Thermo Fisher Scientific, Waltham, MA, USA) and maintained in an incubator at 37 °C with 5% CO_2_. Once approximately 80% confluency was reached, the culture medium was removed and replaced with conditioned medium. The cells were further incubated under the same conditions. After 24 h, the conditioned medium containing extracellular vesicles (EVs) was collected. To remove cell debris, the collected medium was centrifuged, filtered through a 0.22 μm membrane and ultrafiltration column (Millipore, Burlington, MA, USA). The size distribution of EVs was then analyzed using nanoparticle tracking analysis (NTA) [63].

#### 2.2.3. ELISA for the Detection of EV Markers CD63, CD81, TSG101, and Negative Marker Calnexin

The characteristic extracellular vesicle (EV) markers, including CD63, CD81, and TSG101, as well as the negative marker Calnexin, were quantified using enzyme-linked immunosorbent assay (ELISA). The following antibodies were employed: anti-human CD63 rabbit antibody (Cat # MA5-30187), anti-CD81 antibody (Cat # MA5-33123), anti-TSG101 antibody (Cat # MA5-35689), anti-Calnexin antibody (Cat # MA5-35588), and goat anti-rabbit IgG H&L HRP-conjugated secondary antibody (ab6721) [64].

A 96-well microplate was coated with 100 µL of lysed EV samples, diluted in PBS and prepared using a non-SDS lysis buffer. The plate was incubated overnight at 4 °C. After incubation, the wells were washed three times with PBS containing 0.05% Tween-20 (PBS-T), then blocked with 1% bovine serum albumin (BSA) in PBS for 1 h at room temperature to minimize non-specific binding [64].

Following the blocking step, 100 µL of primary antibodies specific to the EV markers CD63, CD81, TSG101, and the negative marker Calnexin were added to the wells and incubated at 37 °C for 1 h. The wells were subsequently washed with TPBS and PBS. After an additional washing step, the HRP-conjugated secondary antibody was added and incubated for 30 min. Color development was performed using TMB substrate, and the reaction was terminated after 10 min by adding 2N H_2_SO_4_. Absorbance was measured at 450 nm using a microplate reader [64].

The OD450 values were corrected by subtracting the background (blank) and expressed as mean ± standard deviation (SD) from three independent EV batches, each measured in technical triplicates. The presence of positive markers (CD63, CD81, TSG101) and the minimal signal for the negative marker (Calnexin) confirmed the identity and purity of the isolated EVs.

#### 2.2.4. Evaluation of the Effects of EVs on MCF-7 Breast Cancer Cells

Once MCF-7 cells reached approximately 90% confluency, they were seeded into 96-well plates at a density of 2 × 10^4^ cells per well. The cells were cultured in DMEM supplemented with varying concentrations of EVs: 0%, 10%, 20%, 40%, 60%, and 80%, while a control group was cultured in pure DMEM supplemented with varying concentrations of EVs collection medium: 0%, 10%, 20%, 40%, 60%, and 80%. The samples were randomly assigned to wells using Excel’s randomization function and all experiments were repeated three times independently.

#### 2.2.5. MTT Assay for Cell Viability

After 48 h of incubation, cell viability was assessed using the MTT (Methylthiazol Tetrazolium) assay. The assay was performed according to the manufacturer’s instructions, and the optical density (OD) was measured at 570 nm using a Biotek Synergy HT Multi-Mode Microplate Reader (BioTek Instruments, Winooski, VT, USA). The assay is based on the ability of viable cells to reduce MTT (3-(4,5-dimethylthiazol-2-yl)-2,5-diphenyl tetrazolium bromide) (Merck Life Science, Darmstadt, Germany) to purple formazan crystals via mitochondrial dehydrogenase activity. The quantity of formazan formed is directly proportional to the number of metabolically active cells. A higher OD indicates greater cell viability, while a lower OD reflects reduced viability. All data analyses were performed under blinded conditions by independent analysts.

#### 2.2.6. Calculation of Cell Viability

The percentage of cell viability was calculated using the following formula:

$$\%\;Cell\;Viability=\frac{{OD}_{570nm\;sample}-{OD}_{570nm\;blank}}{{OD}_{570nm\;control}-{OD}_{570nm\;blank}}\times 100\%$$

where:

Control sample: Contains only cells and culture medium.
Test sample: Contains cells cultured with EVs at different concentrations.
Blank: Contains only the culture medium (without cells).

#### 2.2.7. Scratch Wound Assay

A scratch wound assay was conducted to evaluate the effect of adipose-derived stem cell extracellular vesicles (ADSC-EVs) on the migratory capacity of MCF-7 cells [65]. A sterile pipette tip was employed to create a straight linear scratch across the monolayer in each culture well. Following scratch formation, the wells were rinsed with phosphate-buffered saline (PBS) to eliminate detached cells. Fresh DMEM, either supplemented with ADSC-EVs or without EVs (control), was subsequently added to the wells. Cell migration into the scratch area was monitored and imaged at 0, 24, and 48 h using a CELENA X digital imaging microscope (Logos Biosystems, Anyang-si, Gyeonggi-do, South Korea). The width of the scratch at each time point was measured using the ImageJ software (version 1.53t; National Institutes of Health, Bethesda, MD, USA; https://imagej.net/ij/download.html Accessed 10 October 2025). Calculate the rate of migration as the percentage of wound closure over time [66]:

$$Rate\;of\;migration\;(\%)=\frac{Ao-At}{Ao}\times 100\%$$

where: Ao: Initial wound width at time 0; A_t_: Wound width at time t.

A higher value indicates faster wound healing.

#### 2.2.8. Evaluation of the Effects of EVs on Gene Expression Related to the Cytoskeleton and Signaling Pathways

RNA Extraction and cDNA Synthesis: Total RNA was extracted from MCF-7 cells treated with ADSC-derived EVs using the easy-BLUE™ Total RNA Extraction Kit (iNtRON Biotechnology, Seongnam-si, Gyeonggido, Republic of Korea), according to the manufacturer’s protocol. Following RNA isolation, reverse transcription was carried out using the Maxime™ RT PreMix Kit (iNtRON Biotechnology, Seongnam-si, Gyeonggido, Republic of Korea) to synthesize complementary DNA (cDNA) from the extracted RNA. A total reaction volume of 20 µL was prepared for each sample. The thermal cycling conditions used for cDNA synthesis included incubation at 45 °C for 60 min followed by enzyme inactivation at 95 °C for 5 min.

Real-Time PCR and Gene Expression Analysis: Quantitative real-time polymerase chain reaction (qRT-PCR) was performed to analyze the expression of selected genes associated with cytoskeletal organization and signaling pathways. A total volume of 20 µL per reaction was prepared using the FastGene 2× IC Green qPCR Universal Mix Kit (NIPPON Genetics EUROPE, GmbH, Düren, Germany). Each reaction contained 2 µL of cDNA template and primers at a final concentration of 400 nM. The amplification protocol included an initial incubation at 55 °C for 10 min, followed by DNA denaturation at 95 °C for 1 min. Subsequently, 45 amplification cycles were carried out, consisting of denaturation at 95 °C for 10 s, followed by annealing and extension at 60 °C for 30 s. Melting curve analysis was performed over a temperature range of 60 °C to 95 °C to verify the specificity of the amplified products. SYBR Green was employed as the intercalating dye to detect double-stranded DNA during amplification.

The specific primer sequences used for target gene amplification are presented in Table 1. Glyceraldehyde 3-phosphate dehydrogenase (GAPDH) was utilized as the internal reference gene to normalize gene expression levels across samples.

The expression levels of the target genes were analyzed using the ∆∆Ct (delta-delta Ct) method, as developed by Livak [72]. This method is based on the relative quantification of gene expression normalized to a reference gene—Glyceraldehyde 3-phosphate dehydrogenase (GAPDH) in this study. The following calculations were performed:

$$∆Ct=Ct_{target}-Ct_{reference}$$

$$∆∆Ct=∆Ct_{sample}-∆Ct_{control}$$

$$Fold\;change=2^{-∆∆Ct}$$

where:

- ${Ct}_{target}$: the Ct value of the target gene sample.
- ${Ct}_{reference}$: the Ct value of the reference gene sample.
- ${∆Ct}_{sample}$: ∆Ct of the test sample.
- ${∆Ct}_{control}$: ∆Ct of the reference sample.

This calculation reflects the relative increase or decrease in target gene expression in the experimental group compared to the control.

#### 2.2.9. ELISA for Determination of IL-6 Content

The concentration of IL-6 in the cell culture supernatant was quantified using an enzyme-linked immunosorbent assay (ELISA). Supernatants were stored at −20 °C until analysis. Prior to the assay, samples were centrifuged at 1000 rpm for 3 min to remove residual cellular debris. IL-6 levels were measured using a commercial ELISA kit (Arigo Biolaboratories, Hsinchu, Taiwan) according to the manufacturer’s instructions. Absorbance was recorded at 450 nm using a Synergy HTX microplate reader (BioTek Instruments, Winooski, VT, USA), and each sample was analyzed in duplicate to ensure accuracy and reproducibility.

#### 2.2.10. Analysis of Microtubule Alterations in MCF-7 Cells Following EVs Treatment

MCF-7 cells were cultured to the logarithmic growth phase and seeded at a density of 10^4^ cells per well onto specialized glass-bottom imaging plates. The cells were maintained in DMEM supplemented with 10% FBS and 1% penicillin–streptomycin at 37 °C in a 5% CO_2_ incubator. After 24 h, 20% ADSC-derived EVs were added to the experimental wells, while control wells received EV-depleted medium. Following an additional 24 h incubation, the culture medium was removed, and the cells were fixed in preparation for imaging.

#### 2.2.11. Fixation and Blocking

The culture medium was removed from each well, and cells were washed with 1× PBS (prepared by dissolving 0.8 g NaCl, 20 mg KCl, 0.178 g Na_2_HPO_4_, and 24.5 mg KH_2_PO_4_ in 100 mL distilled water). Cells were then fixed by incubation with 200 μL of a fixation solution containing 3% paraformaldehyde and 0.1% glutaraldehyde for 10 min. After the fixation solution was discarded, 200 μL of a 0.1% sodium borohydride (NaBH_4_) solution (35 mg NaBH_4_ in 3.5 mL of 1× PBS) was added and incubated for 7 min. The reducing solution was then removed, and the wells were washed three times with 200 μL of 1× PBS. Non-specific binding sites were blocked by incubating the cells with 200 μL of blocking buffer (3% BSA and 0.2% Triton-X) for 20 min at room temperature, followed by removal of the blocking solution.

#### 2.2.12. Fluorescent Immunostaining

For primary antibody staining, 150 μL of anti-α-tubulin antibody (Ab6160, Abcam, Cambridge, MA, USA), diluted 1:300 in blocking buffer, was added to each well and incubated with shaking at room temperature for 30 min. The antibody solution was then discarded, and wells were washed three times with 200 μL of washing buffer (0.2% BSA, 0.05% Triton-X) for 10 min per wash with shaking. Secondary antibody staining was performed by adding 150 μL of anti-Rat IgG (H+L) antibody (Alexa Fluor^®^647, ab150159, Abcam, Cambridge, MA, USA), diluted 1:1000 in blocking buffer, to each well. The plates were covered with aluminum foil and incubated with shaking at room temperature for 30 min. Wells were subsequently washed three times with 200 μL of washing buffer for 10 min each and finally rinsed with 500 μL of 1× PBS. Samples were stored in 500 μL of 1× PBS containing 20 mM NaN_3_ until imaging. Super-resolution imaging was conducted using the Nikon N-STORM single-molecule localization fluorescence microscope (Nikon Instruments Inc., Melville, NY, USA).

#### 2.2.13. Statistical Analysis

Each experiment was independently performed by two teams, and differences were assessed using appropriate statistical tests (*p* < 0.05). All experimental data were analyzed using SPSS version 22 (IBM Corp., Armonk, NY, USA). Statistical significance among groups was determined using one-way analysis of variance (ANOVA), followed by Tukey’s Honestly Significant Difference (HSD) post hoc test for pairwise comparisons and effect sizes (Cohen’s *d*) are reported where relevant. Normality was verified by Shapiro–Wilk test. Data are presented as the mean of three independent experiments, with error bars representing the standard error of the mean (SEM).

## 3. Results

### 3.1. Isolation and Characterization of Extracellular Vesicles (EVs) from Adipose-Derived Stem Cells (ADSCs)

**Figure 2:**
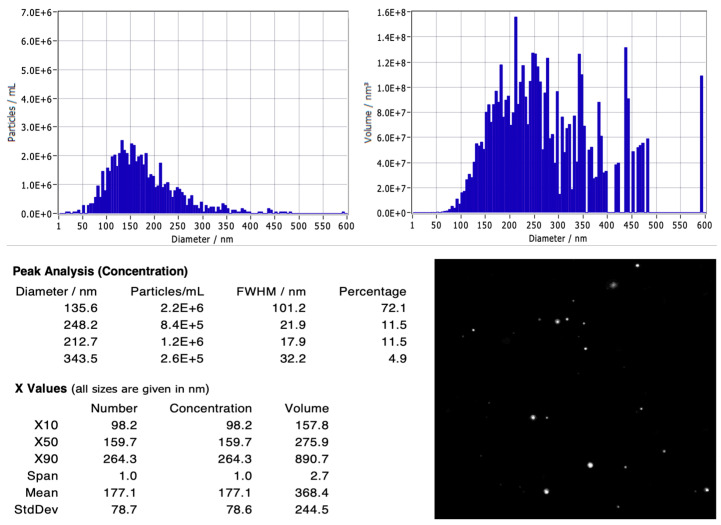
Nanoparticle Tracking Analysis (NTA) of ADSC-derived extracellular vesicles. The analysis was performed on samples diluted 100-fold. The figure displays the particle size distribution relative to concentration and volume, showing a mean particle diameter of 177.1 ± 78.7 nm and a measured peak concentration of 4.49 × 10^6^ particles/mL in the diluted sample. Detailed statistical metrics for the EV population are summarized in the accompanying table.

Figure 2 illustrates the extracellular vesicles (EVs) isolated from the conditioned medium of adipose-derived stem cells (ADSCs) PT-5006 (Lonza, Walkersville, MD, USA), along with their size distribution as analyzed by nanoparticle tracking analysis (NTA). The EVs were diluted 100-fold before measurement. The analysis revealed a relatively uniform size distribution, with an average particle size of 177.1 ± 78.7 nm. The primary peak was observed at 135.6 nm, accounting for 72.1% of the total particles. Additional minor peaks were identified at 212.7 nm and 248.2 nm (each contributing 11.5%) and a small peak at 343.5 nm (4.9%). The size percentiles X10, X50, and X90 were recorded at 98.2 nm, 159.7 nm, and 264.3 nm, respectively, indicating a relatively narrow EV population.

The measured particle concentration at the main peak was approximately 2.2 × 10^6^ particles/mL, decreasing gradually at the minor peaks. Volume-based analysis demonstrated that the majority of EV volume was concentrated in the 150–300 nm range. This is consistent with the known morphological characteristics of Evs, where larger particles, despite being less numerous, contribute significantly to total volume. NTA camera images depicted the Evs as round, bright dots with uniform distribution and Brownian motion, with no evidence of clumping or large contaminants. The total number of Evs was calculated as 4.49 × 10^6^ particles/mL in the 100-fold diluted sample, corresponding to 4.49 × 10^8^ particles/mL in the original sample. These findings confirm that the Evs were isolated with appropriate size and concentration, ensuring sufficient quality and purity for downstream experiments.

#### Dentification of EV Markers by ELISA

Based on the OD450 values obtained from the ELISA (Table 2), the characteristic markers of extracellular vesicles (Evs) were clearly expressed. Among the markers, CD63 showed the highest optical density (1.28 ± 0.12), followed by CD81 (0.96 ± 0.10) and TSG101 (0.54 ± 0.08). In contrast, Calnexin—a negative control marker used to exclude contamination from intracellular organelles—displayed a very low signal (0.05 ± 0.02).

The clear distinction between the positive and negative markers confirms that the isolated particles exhibit the characteristic features of small extracellular vesicles, with minimal contamination by intracellular proteins, thereby ensuring the purity of the EV preparation.

### 3.2. Effect of ADSC-Derived Evs on the Viability of MCF-7 Cells

**Figure 3:**
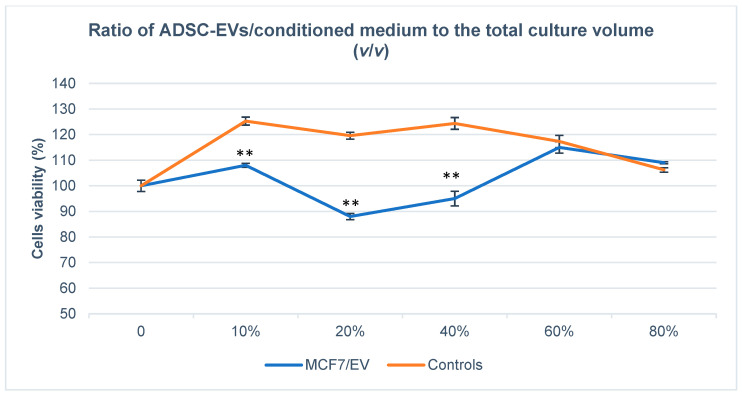
MTT assay results showing the effects of small-sized extracellular vesicles (Evs) derived from adipose tissue-derived stem cells (ADSCs) on MCF-7 cell viability. The graph illustrates cell viability after 48 h of treatment with varying concentrations of ADSC-Evs in conditioned medium (*v*/*v*). A significant reduction in viability was observed at 20% and 40% EV concentrations compared with the control (** *p* < 0.01), with the most pronounced inhibitory effect at 20%. These findings indicate a dose-dependent suppression of breast cancer cell viability by ADSC-Evs.

The viability of MCF-7 cells was assessed using the MTT assay following treatment with either conditioned medium or isolated adipose-derived stem cell–derived extracellular vesicles (ADSC-Evs). Evs were applied at volume ratios of 0%, 10%, 20%, 40%, 60%, and 80% (*v*/*v*) relative to the total culture volume, corresponding to approximate particle concentrations of 0, 4.49 × 10^7^, 9.8 × 10^7^, 1.96 × 10^8^, 2.94 × 10^8^, and 3.92 × 10^8^ Evs/mL, respectively. As shown in Figure 3, treatment with isolated ADSC-Evs significantly affected MCF-7 cell viability in a concentration-dependent manner. A marked reduction in cell viability was observed at the 20% EV concentration, reaching approximately 88–90% of the control level, with statistical significance (*p* < 0.01). This concentration exhibited the strongest inhibitory effect among all tested EV doses. In contrast, treatment with the conditioned medium alone resulted in increased MCF-7 cell viability at concentrations ranging from 10% to 40%, suggesting the presence of soluble factors capable of promoting cell proliferation. At higher EV concentrations (≥40%), the inhibitory effect of ADSC-Evs was progressively attenuated, and cell viability gradually returned toward baseline levels, indicating a biphasic cellular response to EV treatment. Based on these results, a 20% concentration of ADSC-Evs was selected for subsequent experiments, as it consistently produced the most pronounced inhibitory effect on MCF-7 cell viability.

### 3.3. Inhibition of MCF-7 Cell Migration by ADSC-Evs

**Figure 4:**
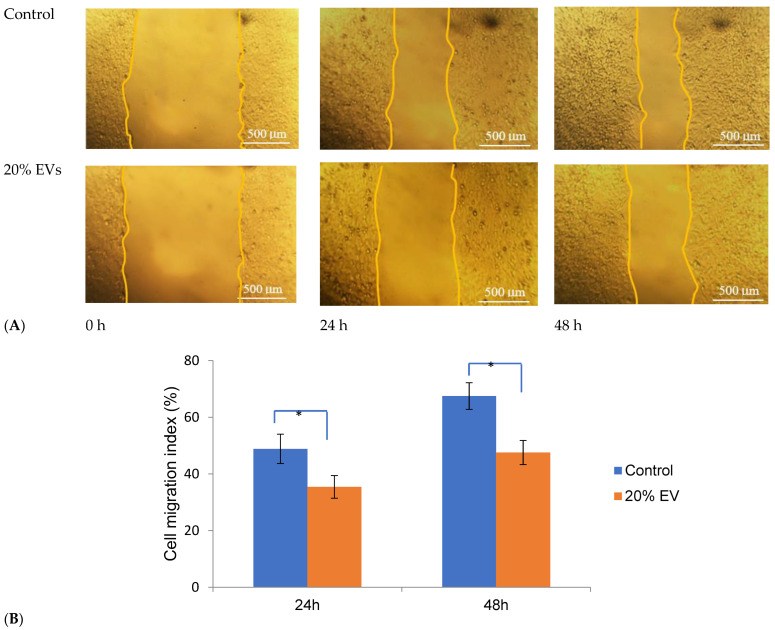
Scratch wound assay assessing the effect of ADSC-derived extracellular vesicles (ADSC-EVs) on MCF-7 cell migration. (**A**) Representative micrographs showing wound closure in control and 20% EV-treated groups at 0, 24, and 48 h. (**B**) Quantitative analysis of wound closure percentage (%) in each group after 24 and 48 h. The control group demonstrated high migratory capacity, with wound closure rates of approximately 48 ± 4.6% at 24 h and 67 ± 4.2% at 48 h. In contrast, cells treated with 20% ADSC-EVs exhibited markedly reduced wound closure, with values of 35.4 ± 3.80% and 47.6 ± 4.2% at 24 and 48 h, respectively, indicating strong inhibition of cell migration. The differences between the control and EV-treated groups were statistically significant (* *p* < 0.05), confirming the potent anti-migratory effect of ADSC-EVs on MCF-7 breast cancer cells.

The inhibitory effect of ADSC-derived extracellular vesicles (ADSC-Evs) on MCF-7 cell migration was evaluated using a scratch wound assay (Figure 4). The control group showed rapid wound closure, with the wound area reach 48 ± 4.6% and 67 ± 4.2% at 24 and 48 h, respectively. In contrast, after 24 and 48 h of treatment, a marked reduction in wound closure was detected in the 20% EV-treated group, with wound closure rates of 35.4 ± 3.80% at 24 h and 47.6 ± 4.2% at 48 h, indicating a significant inhibition of cell migration (*p* < 0.05). These findings demonstrate that ADSC-EV treatment effectively suppresses the migratory capacity of MCF-7 breast cancer cells in vitro.

### 3.4. Expression of TubA1 and CALR Genes Following ADSC-EV Treatment

The analysis of gene expression at the RNA level revealed that ADSC-derived extracellular vesicles (ADSC-EVs) significantly modulated the expression of genes associated with the cytoskeletal framework. TubA1, which encodes α-tubulin, showed a pronounced downregulation with a ΔΔCt value of 7.56, corresponding to an approximately 189-fold reduction in expression (fold change = 0.0053, *p* < 0.05). This marked suppression of TubA1 indicates substantial disruption of the microtubule architecture in MCF-7 cells. CALR, which encodes calreticulin—a protein involved in immune regulation and apoptosis—was also downregulated, though to a lesser extent, with a ΔΔCt of 1.12 (fold change = 0.4605, *p* < 0.05), representing an approximately 2.2-fold decrease in expression. Collectively, these results suggest that ADSC-EVs markedly inhibit the expression of TubA1 and CALR, contributing to the reduced viability and migration of MCF-7 breast cancer cells (Table 3).

### 3.5. Quantification of IL-6 Secretion by MCF-7 Cells Following ADSC-EV Treatment

**Figure 5:**
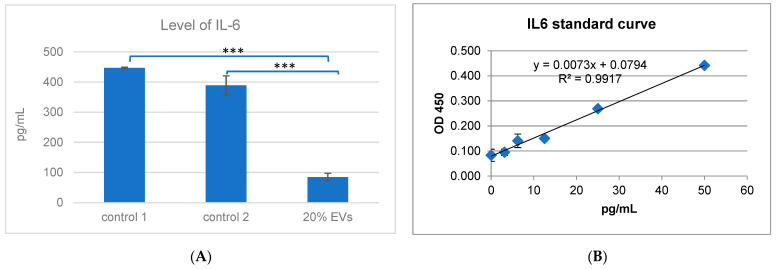
Quantification of IL-6 levels in MCF-7 cell culture supernatants using an ELISA. (**A**) IL-6 concentrations measured in three experimental groups: Control 1, MCF-7 cells cultured in 20% EV collection medium (EV-depleted medium); Control 2, MCF-7 cells cultured in standard DMEM; and Sample, MCF-7 cells treated with 20% ADSC-derived EVs. A significant decrease in IL-6 secretion was observed in the EV-treated group compared with both controls (*** *p* < 0.001); (**B**) Standard curve for IL-6 quantification generated using serial dilutions of IL-6 standards (0–1000 pg/mL), with a correlation coefficient R^2^ ≥ 0.98 confirming assay linearity and accuracy.

The secretion of interleukin-6 (IL-6) by MCF-7 cells following treatment with ADSC-derived EVs was measured using ELISA. As shown in Figure 5, treatment with 20% EVs resulted in a significant decrease in IL-6 levels, from 389.12 pg/mL in the control group to 84.95 pg/mL in the EV-treated group. This reduction in IL-6 secretion was statistically significant (*p* < 0.001). In the two control groups (control 1 and control 2), high levels of IL-6 secretion were observed, measured at 447.07 ± 2.33 pg/mL and 389.12 ± 31.44 pg/mL, respectively. However, in MCF-7 cells treated with 20% ADSC-derived extracellular vesicles (EVs), IL-6 secretion was markedly reduced to 84.95 ± 12.28 pg/mL. This substantial decrease in IL-6 concentration suggests that ADSC-EVs may exert anti-inflammatory or tumor-suppressive effects on MCF-7 cells. These findings indicate that IL-6 expression in MCF-7 cells may be regulated by ADSC-EVs, potentially through modulation of the tumor microenvironment, thereby supporting their therapeutic potential in cancer treatment.

### 3.6. Gene Expression in the IL-6 Signaling Pathway Following EVs Treatment

**Figure 6:**
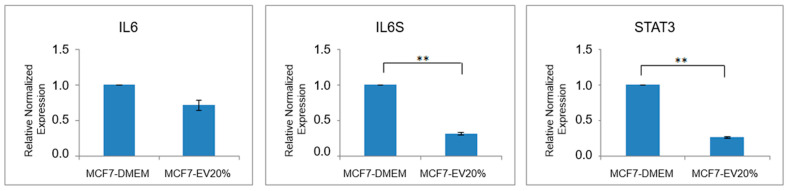
Expression of genes involved in the IL-6 signaling pathway in cultured MCF-7 cells. The bar chart illustrates the relative expression levels of *IL6*, *IL6RST*, and *STAT3* in MCF-7 cells treated with 20% ADSC-derived extracellular vesicles (ADSC-EVs) compared with the control group (MCF-7 cultured in DMEM). A slight decrease was observed in *IL6* expression, while *IL6RST* and *STAT3* were significantly downregulated (** *p* < 0.01). These findings indicate that ADSC-EVs markedly suppress the IL-6/STAT3 signaling axis, which plays a key role in breast cancer cell survival, proliferation, and migration, thereby contributing to the inhibitory effects of EVs on MCF-7 cells.

Quantitative PCR was conducted to assess the expression levels of three key genes within the IL-6 signaling pathway: IL-6, IL-6 receptor subunit beta (IL-6RST), and signal transducer and activator of transcription 3 (STAT3). Figure 6 shows the responses of MCF-7 cells to treatment with 20% ADSC-EVs compared to the control condition. In the control group, gene expression was normalized to a baseline value of 1 for all three genes. In MCF-7 cells treated with ADSC-EVs, IL-6 mRNA expression showed a mild decrease, with a fold change of 0.711 ± 0.068, indicating no statistically significant difference compared with the control group. In contrast, IL-6RST expression was markedly reduced by more than sixfold (fold change = 0.155 ± 0.02, *p* < 0.01). Similarly, STAT3 expression was significantly downregulated in EV-treated cells, showing a 3.87-fold decrease (fold change = 0.258 ± 0.012, *p* < 0.01). These findings suggest that ADSC-EVs selectively suppress components of the IL-6/STAT3 signaling axis, particularly downstream targets such as IL-6RST and STAT3, thereby contributing to their inhibitory effects on MCF-7 breast cancer cells.

### 3.7. Effects of ADSC-EVs on Microtubule Stability

**Figure 7:**
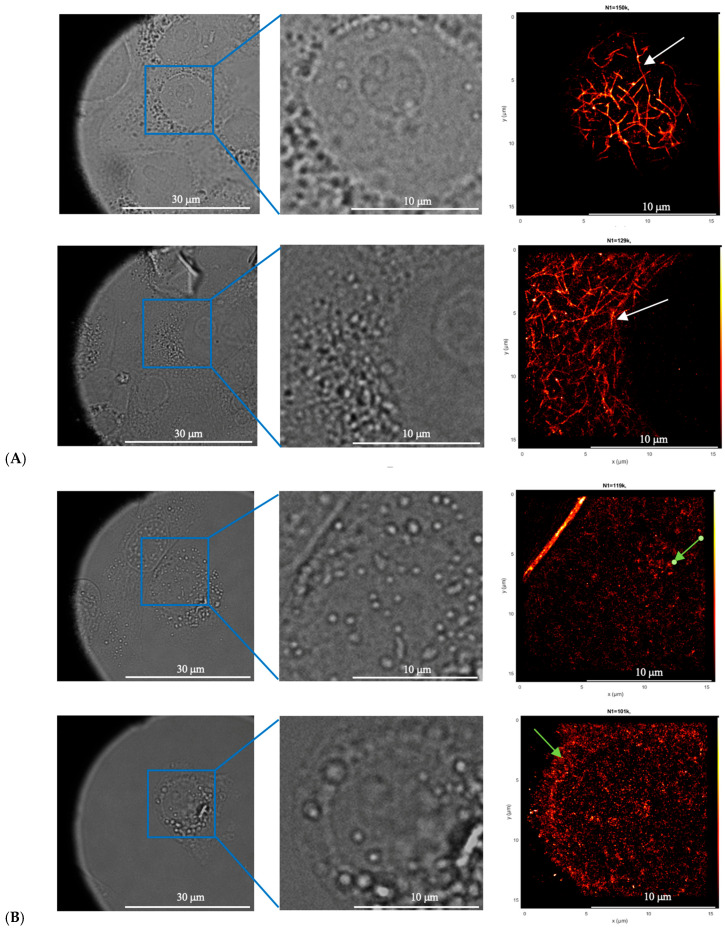
Immunofluorescence images illustrating the microtubule structure in MCF-7 breast cancer cells. Microtubules are stained red with α-tubulin fluorescence, comparing untreated control cells (**A**) with cells treated with 20% ADSC-derived extracellular vesicles (ADSC-EVs) (**B**). In panel (**A**), microtubules appear well-organized, evenly distributed, and form an intact filamentous network (uniform bright red filaments in the fluorescence image—white arrow), indicating a stable and healthy cytoskeletal structure. In contrast, panel (**B**) shows MCF-7 cells treated with 20% ADSC-EVs, where microtubules are markedly disrupted, appearing fragmented, discontinuous, and dispersed into punctate aggregates (irregular red fluorescence—green arrow). The gray background represents transmitted light images showing overall cell morphology. These observations demonstrate that ADSC-EVs induce significant destabilization and damage of the microtubule network in MCF-7 cells, contributing to the observed reduction in cell viability, migration, and proliferation.

The structural integrity of microtubules in MCF-7 cells was examined following treatment with ADSC-derived EVs, and the results are presented in Figure 7. In untreated control cells, a well-organized microtubule network was observed, characterized by strong fluorescence signals, normal cellular morphology, and uniform distribution of alpha-tubulin throughout the cytoplasm. In contrast, in MCF-7 cells exposed to 20% ADSC-EVs, substantial disruption of the microtubule architecture was detected. Fluorescence signals appeared fragmented and irregular, indicating disassembly of the microtubule network. The spatial distribution of microtubules was discontinuous, and structural alterations were evident, suggesting that microtubule stability had been compromised (Figure 7).

In EV-treated samples, alpha-tubulin proteins displayed a disorganized arrangement, lacking the interconnected structure observed in the control group. This disruption in cytoskeletal organization may be attributed to the regulatory effects of ADSC-EVs on microtubule-associated proteins. Such alterations are likely to impair essential cellular processes, including migration, intracellular transport, and mitosis, ultimately contributing to reduced cell viability and apoptosis. These observations support the hypothesis that ADSC-EVs modulate cytoskeletal dynamics as part of their anti-cancer activity.

## 4. Discussion

The present study investigated the effects of extracellular vesicles derived from adipose-derived stem cells (ADSC-EVs) on the behavior of MCF-7 breast cancer cells, focusing on cell viability, migration, inflammatory signaling, and cytoskeletal organization. ADSCs are known to secrete a wide range of cytokines, growth factors, and extracellular vesicles that contribute to tissue repair and immune modulation [73]. ADSC-EVs have been increasingly recognized as key mediators of these paracrine effects, with potential advantages over cell-based therapies due to their stability and safety profile.

The EV population isolated in this study consisted mainly of vesicles within the expected size range for exosomes and ectosomes (microvesicles), which is consistent with previous reports describing the heterogeneity of ADSC-EVs [74,75,76,77,78]. These EVs were confirmed to express canonical exosomal markers (CD63, CD81, and TSG101) while showing negligible levels of the intracellular marker Calnexin, supporting their purity and suitability for downstream experiments.

In terms of functional effects, ADSC-EVs exhibited a concentration-dependent influence on MCF-7 cells. While high concentrations of EV-containing conditioned medium slightly promoted MCF-7 proliferation—consistent with earlier studies showing that ADSC secretome contains proliferative factors [79]—direct treatment with isolated EVs at a moderate concentration (20%) resulted in reduced viability and markedly impaired migration. These findings partially align with Felthaus et al. [80], although differences in ADSC sources and culture conditions may explain the variation in responsiveness across studies. The observation that EVs exerted their strongest inhibitory effect at an intermediate concentration highlights the complexity of EV–cancer cell interactions and suggests that distinct EV subpopulations or cargo components may differentially regulate cancer cell behavior.

Regarding cytoskeletal regulation, previous studies have reported that exosomes can carry cytoskeletal proteins such as actin and tubulin, although direct evidence for EV-mediated regulation of TubA1 expression remains limited [66]. In the present study, downregulation of TubA1 and CALR mRNA was observed following EV treatment. While this does not establish a causal relationship, the changes correspond with the disrupted microtubule organization visualized by immunofluorescence. Notably, CALR is known to participate in immune recognition and stress responses in MCF-7 cells, suggesting that ADSC-EVs may influence multiple cellular pathways beyond the cytoskeleton.

The role of IL-6 in breast cancer biology is highly nuanced and strongly dependent on cellular context. Prior studies have described divergent functions of IL-6, ranging from growth-inhibitory activity in certain breast cancer cell lines [61,81,82] to growth-promoting or drug-resistance-associated effects in MCF-7 cells [83]. In the present study, treatment with ADSC-EVs led to a marked reduction in IL-6 secretion accompanied by decreased expression of IL-6RST and STAT3. These findings are consistent with earlier reports indicating that attenuation of IL-6/STAT3 signaling can impair MCF-7 cell function [61,82] and align with observations from Honma et al. [83]. Collectively, these results underscore the plasticity of IL-6 signaling, which is shaped by receptor availability, intracellular signaling dynamics, and interactions with parallel pathways. Such complexity may explain the diverse biological outcomes attributed to IL-6 across different breast cancer models.

Although EV-mediated suppression of IL-6/STAT3 signaling coincided with microtubule disorganization, it remains unclear whether one process directly drives the other. Wareham et al. [62] previously described IL-6-dependent interactions between STAT3 and stathmin that influence microtubule stability in neuronal systems, raising the possibility of a similar regulatory axis in cancer cells. Nonetheless, additional protein-level analyses and mechanistic studies would be required to verify whether ADSC-EVs act through this pathway in MCF-7 cells. Given that EVs contain a diverse array of proteins, lipids, and RNAs [84,85], multiple components may collectively contribute to the observed phenotypic effects.

This study has several limitations. First, the experiments were conducted on a single estrogen-receptor-positive cell line, and the effects of ADSC-EVs may differ across molecular subtypes of breast cancer. Second, gene expression was evaluated at the mRNA level only; future studies should include Western blotting or proteomics to validate protein-level changes in IL-6/STAT3 signaling and cytoskeletal regulators. Third, the specific EV cargoes responsible for the observed changes remain unidentified. Comprehensive profiling, such as EV proteomics or small RNA sequencing, would help clarify the mechanisms underlying the anti-proliferative and anti-migratory effects observed in this study.

Overall, the findings suggest that ADSC-EVs can modulate breast cancer cell behavior by influencing inflammatory signaling pathways and cytoskeletal organization. While the results provide preliminary insight into their potential anti-tumor activity, further investigation is necessary to determine the specific EV components responsible and to assess the translational relevance of these observations across different breast cancer models.

## 5. Conclusions

The impact of adipose-derived stem cells (ADSCs) on the growth and metastasis of breast cancer cells has been increasingly investigated in recent years. It has been reported that ADSCs can exert either promotive or inhibitory effects on the development of breast cancer cells (BCCs), depending on the context. Extracellular vesicles (EVs) isolated from ADSC cultures were characterized by a concentration of 4.49 × 10^8^ particles/mL, with an average particle size of 177.1 ± 78.7 nm. These EVs were found to inhibit the proliferation of MCF-7 breast cancer cells, with the most pronounced inhibitory effect observed at a concentration of 20% (corresponding to 9.8 × 10^7^ particles/mL). In addition, ADSC-derived EVs significantly reduced IL-6 expression at both the protein and mRNA levels and downregulated the mRNA expression of IL-6 receptor (IL-6R) and STAT3. At the same 20% concentration, EV treatment was also associated with marked structural disruptions in the microtubules of MCF-7 cells, suggesting a potential link between cytoskeletal disorganization and impaired cell viability.

## Figures and Tables

**Table 1:** Primers used for RT-PCR verification.

Primer	Sequence (5′–3′)	Size (bp)
TubA1	F: CGGGCAGTGTTTGTAGACTTGG R: CTCCTTGCCAATGGTGTAGTGC	102 bp [67]
IL-6	F: AGACAGCCACTCACCTCTTCAG R: TTCTGCCAGTGCCTCTTTGCTG	132 bp [68]
STAT3	F: CTTTGAGACCGAGGTGTATCACC R: GGTCAGCATGTTGTACCACAGG	133 bp [69]
IL-6ST	F: CACCCTGTATCACAGACTGGCA R: TTCAGGGCTTCCTGGTCCATCA	134 bp [70]
GAPDH	F: TTGTCTCACTTGTTCTCT R: ATGGGAGTTGTTTTCTTG	87 bp [71]

**Table 2:** ELISA results for the identification of EV markers.

Marker	OD450	SEM	*p*-Value
CD63	1.28	0.12	*p* < 0.001
CD81	0.96	0.1	*p* < 0.001
TSG101	0.54	0.08	*p* < 0.01
Calnexin	0.05	0.02	

**Table 3:** qPCR Results for the TubA1 and CALR Gene.

Gene	ΔΔCt	Fold Change	SEM	*p*-Value
TubA1	7.56	0.0053	0.0002	0.019
CALR	1.12	0.4605	0.0022	0.0237

## Data Availability

Data are available upon reasonable request. Please contact the corresponding author.
